# Development of a gratitude intervention model and investigation of the effects of such a program on employee well-being, engagement, job satisfaction and psychological capital

**DOI:** 10.3233/WOR-220604

**Published:** 2025-03-18

**Authors:** Bo Harty, John-Anders Gustafsson, Monica Thorén, Anders Möller, Ann Björkdahl

**Affiliations:** a Avonova Management Support, Gothenburg, Sweden; b City of Trollhättan, Trollhättan, Sweden; c Gothenburg University, Institute of Medicine, School of Public Health and Community Medicine, Gothenburg, Sweden; d Gothenburg University, Sahlgrenska Academy, Institute for Neuroscience and Physiology, Rehabilitation Medicine, Gothenburg, Sweden

**Keywords:** Gratitude, group intervention, psychological capital, health, training the trainer

## Abstract

**BACKGROUND:**

In a demanding working life, it is important to determine how individuals can thrive at work. In a previous study we investigated whether a program of gratitude interventions can increase psychological wellbeing, engagement, job satisfaction, and psychological capital showing promising results.

**OBJECTIVE:**

The present study aimed to present the development of a manager coached group intervention program related to gratitude at workplaces and investigate the effects of such a program on the same variables.

**METHODS:**

The intervention included five group sessions of gratitude dialogue between employees, supervised by their first line managers. Participants were assigned to an intervention or control group. Assessments were made before and after the intervention program and followed-up at 6 months post-intervention. Both quantitative and qualitative analyses were performed. Both groups completed instruments measuring positive psychological capital (PCQ), work engagement (UWES), psychological wellbeing (PGWB-S), and job satisfaction (aJDI). All managers were interviewed after the intervention.

**RESULTS:**

Compared with the control group the gratitude dialogue intervention was found to significantly enhance psychological wellbeing, engagement, and job satisfaction. The results were supported by the interviews with managers.

**CONCLUSION:**

The results suggest that gratitude dialogues at work may be an effective way of improving employee wellbeing. Suggestions on how to improve the results from this kind of gratitude intervention further are presented.

## 1 Introduction

In the current rapidly changing society, organisations face immense challenges related to for example employment conditions, political claims, profitability and globalisation. To be relevant both in the present and in the future, organisations must be able to change quickly, be innovative, and create workplaces in which employees are engaged and can be at their best [1]. In a global economy, it is a challenge for today’s organizations to motivate people and get them to stay at work. Given this challenge, researchers see an increasing need to promote strong employee engagement in the workplace [2]. A crucial question is how to generate the best conditions under which employees can thrive, such as employee engagement, health, job satisfaction, and psychological capital. In a systematic Review and Meta-Analysis of positive psychology interventions Donaldson et al. [3] found employee gratitude and strengths interventions to have stronger mean effects on desirable work outcomes, than other interventions.

In an earlier study [4], we found that gratitude interventions (exercises in focusing aspects of life that are worth being grateful for) can encourage positive developments in self efficacy, job satisfaction and positive emotions. The study from 2016 was performed at few workplaces. The question arose if the model can be transformed to a larger number of places by letting the groups be coached by their first line managers. In the present study, we investigated this subject further by enlarging the study group and by letting first line managers, after training, instead of professional psychologists as in the above mentioned previous study, lead the intervention groups.

### 1.1 What is gratitude?

Gratitude interventions are regarded as one of the most powerful tools in the field of positive psychology for enhancing well-being [5]. Interest in this topic is increasing among organizational researchers. In fact, over half of the studies done on gratitude (55% ) in organizational behavior (OB) have been published in the last five years [6].

It has been extensively studied both on an individual and, to a growing extent, on an organisational level. [7] defined “the grateful disposition as a generalised tendency to recognise and respond with grateful emotion to the roles of other people’s benevolence in the positive experiences and outcomes that one obtains” (p. 112). Depriest (2022) defines gratitude “as feeling thankful and having a general readiness to show and return appreciation and kindness” (p. 1) [8]. A grateful disposition refers to an individual trait, whereas gratitude as an individual state describes a transitory feeling of gratefulness [9]. In organisations, gratitude is experienced by individual members, and is also perceived in the atmosphere of the organisation. Interpersonal interactions among persons who are grateful create friendly attitudes, values, and behaviour. Gratitude on an organisational level has been conceptualised as collective gratitude and is defined as the shared gratitude between individuals of an organisation [10, 11].

### 1.2 Gratitude and the individual

In a review of the relevant literature, Wood et al. [5] reported that gratitude is related to numerous personality characteristics that contribute to positive social relationships. Gratitude was also found to be strongly associated with mental and physical health, positive emotions, life satisfaction, and well-being. More recent studies have confirmed many of these results [12–16]. According to Wood et al. [5], longitudinal and experimental studies have indicated there to be a causal relationship between gratitude and well-being.

### 1.3 Effects of individual gratitude interventions

In their literature review, Wood et al. [5] examined 12 gratitude intervention studies that used different methods, mainly listing things for which to be grateful or writing gratitude letters, and concluded that gratitude interventions are clearly effective for increasing well-being in individuals. Six years later, Davis et al. [17] performed a meta-analysis of gratitude interventions and reached the same conclusion; they reported that gratitude interventions enhance psychological well-being.

Wong et al. [18] reported that a gratitude intervention decreased psychological distress and increased gratitude, satisfaction, and meaning in life. Lai and Carroll [14] found that a gratitude intervention increased gratitude, and induced less negative and more positive emotions. Psychotherapy clients who completed gratitude exercises have been reported to have a better mental health than controls [19]. In a literature review with focus on experimental and longitudinal studies Jans-Beken et al. [20] examined the causal relationships between gratitude and human health. They found inconclusive results regarding the effect of gratitude on physical health and psychopathology. On the other hand, their review showed that gratitude facilitates emotional and social well-being. Relatively modest effects of gratitude interventions were found on depression and anxiety in a meta-analysis by Cregg and Cheavens [21]. In a systematic review of 19 studies of gratitude interventions and effects on physical health mixed results were found [22]. Subjective sleep quality was improved. Blood pressure, glycemic control, asthma control and eating behavior also demonstrated improvements although they were understudied. Among people with low to moderate levels of well-being and moderate distress Bohlmeijer et al. [23] conducted a 6-week gratitude intervention and the results indicated an enhanced mental well-being but not distress at post-test, 6 weeks and 6 months follow-up.

### 1.4 Gratitude and organizations

These results on gratitude in individuals could also have implications for organisational settings. Although scientific work on gratitude in organizations exists they are comparatively less than studies on individual gratitude [6]. Indeed, there has been a growing interest in the impact of gratitude on the life of organisations in recent years. In a meta-analytic review of prosocial behaviour, Ma et al. [24] found a clear connection between gratitude and prosocial behaviour. Gratitude has also been reported to be related to organisational citizenship behaviour, that is employee behaviour important for the organisation although it isn’t the individual’s main work task [25, 26] and a greater sense of responsibility towards employee and societal issues [27]. Similarly, gratitude is reportedly associated with a better job satisfaction [28–30], as well as less burnout and exhaustion, more psychological well-being, and fewer absences due to illness [29–31]. Gratitude also strengthens interpersonal relationships [32, 33] and creates trust between individuals [34]. In a review on the construct of gratitude, Di Fabio et al. [35], stated that “gratitude seems crucial for employees’ efficiency, success, productivity, and well-being” (p. 4). In a study of 1187 workers from 72 organizations Komase et al. [36] found a significant positive association between collective gratitude and work engagement.

### 1.5 Effects of organizational gratitude interventions

Although research on gratitude has resulted in promising outcomes for using gratitude interventions in organisations, studies on this subject are still lacking. In one study, Kaplan et al. [37] implemented gratitude interventions in a workplace context. Employees completed self-guided gratitude journaling of work-related experiences during a two-week period. The authors found significant increases in well-being and gratitude, as well as a reduction in workplace absence due to illness. After an 8-week gratitude intervention program (gratitude journaling), school teachers showed increased life satisfaction and more positive affect [38]. Using the same methodology, Chan [39] found that a gratitude intervention improved life satisfaction and the sense of personal accomplishment, together with a decrease in emotional exhaustion. Cheng et al. [40] implemented a gratitude intervention in which participants were asked to journal their work-related gratitude twice a week over 4 consecutive weeks among health care practitioners. They found that this intervention resulted in lower depressive symptoms and perceived stress post intervention and at the 3-month follow-up. In a review Komase et al. [41] studied the effects of gratitude intervention on mental health and well-being among workers and found a significant improvement in perceived stress and depression; however, the effects on well-being were inconsistent.

A slightly different approach was used by Harty et al. [4] in an 8-week program in which employees participated in exercises that focused on gratitude connected to their daily work biweekly, as a group. Compared with control participants, the gratitude group exhibited a greater level of positive emotions, self-efficacy, and job satisfaction. These positive changes persisted 6 months after the intervention. In multiple gratitude interventions implemented over 1 year, Stegen and Wankie [42] found improved overall job satisfaction in a school of nursing.

Gratitude interventions are therefore clearly one option to create workplaces in which people are healthy and thrive at work. Collectively, previous work indicates that gratitude helps people to see the world from a more positive perspective, and therefore has the potential to enhance various favourable employee and organisational characteristics.

The most common intervention method in organisations is individual gratitude journaling. In our previous study, we found that participants benefitted from the group dynamics elicited when people shared their work-related gratitude. This story-telling may have benefitted the work environment and resulted in a longer-lasting grateful attitude. However, in the previous study the participants were relatively few (N = 66) and the groups were conducted by two experienced psychologists. We wanted to test the same intervention method on a larger scale, more participants, and make it more available to a wider range of workplaces, and study the effects of the intervention when the groups are coached by their first line managers using the “train the trainer” method, whereby managers receive training to perform gratitude interventions with their own employees.

## 2 Objective

**The aims of the present study are:**

1. To present the development of a manager coached group intervention program related to gratitude at workplaces.
2. To investigate the effects of such a program on employee well-being, engagement, job satisfaction and psychological capital.

## 3 Methods

### 3.1 Intervention program

The intervention program was built by the authors based on the literature presented above and on general positive psychological theories [43] and group development methods. The purpose was to create dialogue and awareness of real gratitude feelings connected to the workplace and in other situations. Another intention was to make an easily implemented and engaging experience so that the content of the intervention can be a useful tool for enhancing gratitude in workplaces. One basic principle is activity – by being active in exercises of different kinds the pleasure and learning are supposed to increase. To our knowledge, there is no similar intervention program described in the literature. The program is described in the result section.

### 3.2 The empirical study of effects

**Fig. 1:**
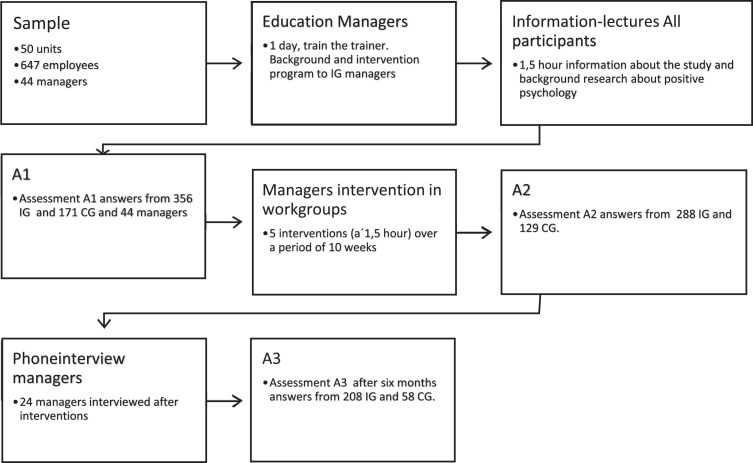
Flowchart showing the study process.

This was a longitudinal intervention study that included an experimental and a control group, and which adopted both quantitative and qualitative analyses. Assessment periods were immediately before the start of the intervention (A1), immediately after the end of the intervention (A2), that is 10 weeks after A1, and six months after end of intervention (A3) (Fig. 1).

#### 3.2.1 Data collection

Data were collected using four well-known instruments that were compiled in a survey using the Questback platform (Netigate.net). In the second assessment, two open-ended questions were added to the questionnaire. Interviews were held with all the 24 managers who oversaw the intervention to obtain qualitative data about their perception of the intervention.

#### 3.2.2 Instruments

Positive Psychological Capital Scale **(PCQ):** We used the **PCQ** [44, 45]. The questionnaire consists of 12 items that are scored on a 6-point Likert-type scale ranging from “strongly disagree” 1 to “strongly agree” 6.

**Utrecht Work Engagement Scale** (UWES-9): The nine-item Utrecht Work Engagement Scale was used to measure vigour, dedication, and absorption to work Schaufeli et al. [46]. The scale consists of nine claims about feeling towards work, each of which is scored on a Likert-type scale (0 never to 6 always). The UWES-9 has been reported to have a good construct validity [47] and a Cronbach’s alpha across countries range between.72 and.90 Schaufel et al. [46].

**Psychological General Well-being-Short** (PGWB-S): The Psychological General Well-being-Short was used to measure perceived health. The PGWB-S [48] is a short version of the PGWB [49]. It measures mood using six questions covering anxiety, depression, well-being, self-control, general health, and vitality. Each question is rated on a six-point Likert-type scale (1 – 6), the higher number the better well-being The instrument has been shown to have good acceptability and validity for its use in various settings and a Cronbach’s alpha ranging between 0.80 and 0.92 [48].

**Abridged Job Descriptive Index (aJDI) – including Abridged Job In General (aJIG):** The Abridged Job Descriptive Index (aJDI [50] is a short, five-item version of the JDI [51]. It measures job satisfaction in five aspects, including work in current workplace, current salary, promotion opportunities, tutorial opportunities, and perception of colleagues. The Abridged Job In General (aJIG) scale [52] is a short version of the JIG [53], and consists of one aspect of the JIG that is scored using eight items; workers overall evaluations of their job. Considerable support for its validity and reliability (Cronbach alpha coefficient.87) has been reported [52].

Efforts were made to find scales that had been translated into Swedish. However, the aJDI needed to be translated into Swedish by the authors. To ensure that the translation was as close as possible to the original English version, we performed a back-translation to English from Swedish [54].

#### 3.2.3 Open-ended questions

At the A2 assessment, the questionnaire included two additional open-ended questions: “Has participation in the project resulted in any changes in your workplace? If so, what? Describe this in your own words” and “How would you describe your experience participating in the meetings in this project? Describe this in your own words”.

#### 3.2.4 Interviews

All managers that participated in the intervention (*n* = 24) were interviewed one month after the intervention period. Interviews were carried out over the telephone, and managers responded to the three following questions in their own words: How would you describe your experience participating in the project? What effects of the interventions have you observed? Is there anything we could have done better to facilitate the intervention for you? The interviews were recorded, and clarification questions were asked if necessary to elucidate any unclear points and glean a deeper understanding of the managers’ opinions. The managers were also asked to quantitatively rate their experiences of participation in the study (from 1 = “not at all positive”, to 5 = “very positive”) and of the effects of the intervention (from – 5 = “very negative effects”, to + 5 = “very positive effects).

#### 3.2.5 Sample

The survey was sent out to 647 employees, intervention (IG, N = 421) and control (CG, N = 226) groups and to 44 managers (IG: *n* = 24; CG: *n* = 20) at the first assessment period (A1). The sample comprised 50 centres (retirements homes, disability accommodations, education units, administrative units, service units, and primary health care centres) within municipality- and county-based organisations in the western part of Sweden. Represented professions were healthcare assistants, preschool teachers, elementary school teachers, adult education teachers, administrators, cleaning and kitchen staff, doctors, nurses, psychologists, physiotherapists, and social workers. After selecting the organisations, their HR department made an internal inquiry to find out which units were interested to participate in the study. We allocated 28 units to the intervention group and 22 centres to the control group. Group sizes ranged from 4 to 33 people. Individual data like gender, age and professional experience was not collected for integrity and practical reasons.

#### 3.2.6 Dropouts

Of the 647 surveys sent to employees at each assessment period, 147 employees (23% ) did not respond at the first assessment. At the second assessment, 230 employees (35% ) did not respond, and at the third assessment 280 employees (43% ) did not respond.

We did not collect information on the reasons for dropping out. When performing longitudinal studies in “real-life” settings, dropout of some participants is inevitable.

To obtain a complete dataset, participants that failed to complete the survey both before and after the intervention were removed, which resulted in a final total of 380 employees. Of the remaining 380 employees, 277 were in the IG and 103 in the CG.

### 3.3 Data analysis

#### 3.3.1 Statistical analysis

The sum scores of each of the instruments were analysed, and parametric statistics were therefore applied. We hypothesised that the intervention would have positive effects on well-being, engagement, job satisfaction, and psychological capital. A mixed model multivariate analysis of variance (MANOVA) was chosen. Preliminary assumption testing was conducted to check for normality, linearity, univariate and multivariate outliers, homogeneity of variance-covariance matrices, and multicollinearity, with no serious violations noted. The MANOVA was performed to test for interaction between intervention/control group and time (A1, A2, A3), including all four instruments, the PGWB-S, UWES, AJDI, and PCQC, as outcome variables. Corrections were made for possible differences between groups at baseline (A1). In case of a significant interaction simple effects were tested using Student’s *t*-test for all the instruments comparing assessments 1-2 and 1–3. Two-sided test was used and *p*-values below 0.05 were considered statistically significant. The effect size (eta) was calculated for results that showed a significant difference (*p* < 0.05). The guidelines [55] for interpretation of the eta value are as follows: small effect = 0.01, moderate effect = 0.06, and large effect = 0.14. Differences between scores at assessments 1-2-3 for the IG and CG were also analysed in groups of the separate work units; service, care of persons with disability and elderly, administrative units, schools and health care units. All analyses were performed using SPSS (version 28).

#### 3.3.2 Qualitative analysis

The answers to the open questions were analysed using descriptive qualitative content analysis [56]. The answers were read through and all meaning units/statements, including expression of valuation of the experience, were identified. These were then categorised into “positive” or “negative” experiences. Subcategories and examples of different answers will be presented in the results section. Given that many participants answered these questions, some quantitative data (frequencies) will also be presented.

The interviews with managers were analysed in the same way as the open-ended questions. The recordings were listened to several times to identify meaning units, which were then categorised. Given that all managers were interviewed and rated their experiences, some quantitative data will also be presented. In the result section the medians (Md) of the interview persons’ ratings of their experiences and of the effects of the intervention are presented.

### 3.4 Ethical considerations

Regarding ethical approval the policy in Sweden is that for developmental work within the organizations no ethical application is needed. The present project was made in cooperation with the employers and human resource departments as a part of their work environment work.

This study followed the Declaration of Helsinki ethical guidelines. All participants received information about the purpose and procedures of the study. Participation was voluntary both for the individuals and the departments.

The aim of the study was to create positive thoughts, and we wanted the interventions to be considered as rewarding.

We used a web-based questionnaire through the Questback platform to collect data. A link to the questionnaire was sent to the E- mail address of each participant. The participants’ privacy was guaranteed.

## 4 Results

### 4.1 Description of the intervention model

The intervention was initiated with a training day for the managers, who would be coaching the interventions in their units. This training day consisted of a theoretical lecture where positive psychology was explained and practical exercises of the different moments in the intervention program. All participants from the IG were invited to informational lectures about the project and its purpose; we described the background and basic research in positive psychology. Then, the first web-based questionnaire was sent out. After this, the managers implemented five, 1.5-hour intervention sessions in their units; there was one intervention session every 2 weeks, resulting in a total intervention period of 8 weeks. After the fifth intervention session, participants completed the web-based questionnaire a second time. After 6 months, the web-based questionnaire was completed for the third time. Throughout the time of intervention the two psychologists, who performed the training program were accessible for discussions and advices to the coaches.

**1^st^ session**

The session started with a short introduction and background for 15–20 minutes from the manager. The manager informed the group about the arrangement of the project, its purpose, who would be involved, and current research on the positive effects of gratitude and positive thinking. The first intervention task was to identify gratitude factors, first individually, then in pairs, and then, when possible, in smaller groups; finally, participants were asked to evaluate and prioritise these gratitude factors in the entire group. Finally, there was a group discussion about how to integrate these factors in participants’ daily life.

**2^nd^ session**

The group chose, prepared, and showcased a creative way to illustrate the gratitude aspects from the previous session, such as a song, poem, picture, or short play. The group then evaluated the last two weeks at work with a focus on gratitude factors. The session ended with a group discussion about how to maintain a positive focus in the workplace.

**3^rd^ session**

In groups of four, one participant was interviewed by the three others on instances in the last two weeks at work that had generated feelings of gratitude. Each person in the rest of the group was then interviewed in the same way. The summaries of the interviews were presented and discussed with the other groups. The session ended with a dialogue about how to create an even more positive focus in the workplace.

**4^th^ session**

On three sheets of paper, each participant wrote down their feelings of gratitude in relation to i) why work is meaningful, ii) why work gives them joy, and iii) how work engages their capabilities. Three subgroups then summarised and prioritised each topic and presented it to the rest of the group. Finally, these topics were discussed in small groups.

**5^th^ session**

This session included an exercise to amplify experiences of gratitude and strengthen gratitude in the workplace. In threes, one participant was interviewed by a second participant, and the third observed the interview. The task of the observer was to note everything that the interviewer did to focus and enhance the experience of gratitude. Each of the three roles was rotated. Finally, there was a small idea-generating contest to find the best ideas for creating a positive focus in the workplace.

### 4.2 Statistical results

A total of 380 participants (IG: *n* = 277; CG: *n* = 103; women: *n* = 300; men: *n* = 80) from 54 units answered the questionnaire both before and after the intervention. The 54 units were public workplaces in two different communes in the west of Sweden. The area of work consisted of care of elderly and disabled (*n* = 154), school and preschool activity (*n* = 94), district health care centres (*n* = 39), service, i.e., cleaning and canteen workers (*n* = 47), and administration (*n* = 46). Table 1 presents descriptive results on the different instruments completed by the two groups.

**Table 1:** Presentation of means, (SD), medians, and range for the instruments PGWB-S, AJDI, UWES and PCQC at the three assessments, before intervention (baseline, A1), after intervention (after 10 weeks, A2) and follow-up, 6 months post intervention (A3)

	Intervention group N = 277			Control group N = 103		
	Baseline A1	After 10 w A2	Follow-up A3	Baseline A1	After 10 w A2	Follow-up A3
**PGWB** *mean (SD)*	75.2 (19.2)	78.6 (18.0)	73.0 (21.6)	74.0 (19.6)	72.3 (19.6)	72.9 (19.4)
*median (range)*	77 (26–110)	80 (29–110)	77 (22–110)	77 (29–110)	73 (22–102)	77 (11–106)
**AJDI** *mean (SD)*	77.9 (15.0)	82.0 (15.7)	78.4 (17.6)	74.9 (16.8)	77.3 (16.5)	79.4 (17.4)
*median (range)*	80 (27–109)	84 (27–111)	82 (25–112)	77 (22–106)	82 (24–108)	82 (24–108)
**UWES** *mean (SD)*	40.1 (8.0)	40.5 (8.6)	38.9 (9.5)	38.2 (9.8)	37.9 (9.7)	38.5 (9.7)
*median (range)*	41 (17–54)	42 (6–54)	41 (6–54)	39 (10–54)	40 (4–54)	41 (1–53)
**PCQ** *mean (SD)*	56.2 (8.4)	57.5 (7.7)	56.1 (8.3)	56.0 (8.4)	56.0 (8.2)	56.1 (7.6)
*median (range)*	57 (25–72)	58 (28–72)	56 (33–72)	57 (24–72)	57 (28–72)	58 (34–71)

Abbreviations: PGWB = Psychological General Well-being; AJDI = Abridged Job Descriptive Index; UWES = Utrecht Work Engagement Scale; PCQ = Positive Psychological Capital Scale.

In the mixed MANOVA, including PGWB-S, UWES, AJDI and PCQ sum scores as dependent variables, we found a significant interaction between treatment group and time (A1, A2, A3) (F = 2,102, *p* = 0.035) (Table 2). In order to investigate these interactions further analyses with student *t*-test was performed for each of the dependent variables, and this showed significance between-group differences after the intervention (at A2), regarding PGWB-S (*p* = 0.004, mean difference 6,261, 95% CI 2.064 – 10.457), AJDI (*p* = 0.011, mean difference 4,694, 95% CI 1.084 – 8.304) and UWES (*p* = 0.032, mean difference 2,582, 95% CI 0.232 – 4.932) scores in favor of the intervention group. Although statistically significant, these differences were small in magnitude (eta squared = 0.02). The between-group comparison of the PCQ score revealed a tendency towards a significant difference. For the other two assessments (A1 and A3), no significant difference between groups was found for any of the four scales, neither at baseline (A1) nor at 6 months follow-up (A3).

**Table 2:** Test of fixed effects group and time. Repeated measure MANOVA

Instrument	F-value	Sign
Combined dependent variables	2,10	*p* = 0,035
PGWB	5,20	*p* = 0,006
AJDI	2,69	*p* = 0,070
UWES	2,44	*p* = 0,089
PCQ	2,00	*p* = 0,136

Abbreviations: PGWB = Psychological General Well-being; AJDI = Abridged Job Descriptive Index; UWES = Utrecht Work Engagement Scale; PCQ = Positive Psychological Capital Scale.

Additional analyses were also made to check the development within different groups of work units (service, care of persons with disability and elderly, administration, schools and health care units) (Table 3). Due to small numbers, in some of the units, the results need to be taken cautiously. This subgroup analysis was not possible to perform with the health care unit as the numbers were too small. These analyses show that there are improvements in some units and not in other. The units with significant differences between groups were service and care of persons with disability and elderly.

**Table 3:** Significant differences between the groups (IG and CG) presented for different areas of work at first and second assessment

Area of work	No. of participants	Instruments A1	*p* > 0.05	Mean diff	CI 95 %	Instruments A2	*p* > 0.05	Mean diff	CI 95 %
Care of elderly and disabled	IG 112 CG 33	UWES	*p* = 0.003	4,935	1.67 – 8.19	PGWB UWES PCQ	*p* = 0.01 *p* = 0.006 *p* = 0.01	9,816 5,034 3,944	2.35 – 17.28 1.50 – 8.57 0.89 – 7.00
School and preschool activity	IG 62 CG 38	No diff	AJDI	*p* = 0.02	6,602	1.05 – 12.51
Service	IG 27 CG 20	PGWB PCQ	*p* = 0.02 *p* = 0.02	13,725 6,587	2.10 – 25.35 0.98 – 12.19	PGWB UWES PCQ	*p* = 0.001 *p* = 0.01 *p* = 0.004	18,286 8,300 8,144	7.98 – 28.58 0.82 – 28.31 2.75 – 13.53
Administration	IG 28 CG 18	No diff	No diff
Primary health care centres	IG 39 CG 0	No analyse	————

Abbreviations: PGWB = Psychological General Well-being; AJDI = Abridged Job Descriptive Index; UWES = Utrecht Work Engagement Scale; PCQ = Positive Psychological Capital Scale.

### 4.3 Qualitative result

#### 4.3.1 Participants' expressed experiences of participation

Of the 380 participants who answered the questionnaire one month after the intervention, 173 people answered the first open ended question (“Has the project contributed to any changes at your workplace? If yes, in what way?”). 183 responded to the second question (“How was your experience participating in the project?”).

Of the 173 participants who answered the first question, 142 reported positive effects and 31 reported no effect of the intervention. Of the 31 that reported no effect, 10 participants reported that their workplace already had a very positive atmosphere. The positive reports were categorised as relevant to emotional, cognitive, and social aspects. The emotional aspect contained statements about increased joy, comfort, well-being, energy, and harmony in the group, and that the interventions were rewarding. The cognitive statements concerned having the time to reflect, to try new thoughts in relation to the daily work process, engaging in interesting and instructive discussions, and experiencing a greater awareness about different kinds of solutions to problems at work, and about their own contribution to social processes at the workplace. Social aspects included reports of an increased mutual helpfulness, better and more open communication, an increased understanding of each other, listening more carefully to each other, an increased sense of community, becoming closer with each other, making more of efforts to see and please each other, and of happiness and satisfaction in the group.

Of the 183 people who answered the second question most (*n* = 162) reported having had a positive experience. A total of 23 people reported having had a negative experience, which means that some people reported both positive and negative experiences. Regarding the positive experiences, participants’ responses were similar to responses to question 1. The positive emotional experiences were expressed using words such as “joy”, “happiness”, and “pleasure”. Cognitive aspects included reports that the exercises were inspiring, that they had learned a lot and gained new insights about both themselves and about others, that their perspectives have been widened, and that they felt stimulated to have a more positive outlook.

Some respondents also reported perceiving the exercises as both joyful and demanding. The negative statements ranged from “I have not gained much from it” to “the experience was draining for me”. There were also reports that the exercises were boring, and non-authentic. Two people reported that the exercises were not intellectually challenging enough.

#### 4.3.2 Coaches’ expressed experiences of coaching the intervention groups

In the interviews with the coaches only positive responses were given to the first question (how have you experienced to participate in the project?). Positive emotional answers included the words “funny”, “nice”, and “positive”, which were mentioned by 22 people. The managers reported the experience to be “instructive”, “interesting”, “useful”, and “inspiring”; these words were mentioned by 23 people. Two people reported that the intervention reinforced the group’s positivity from the beginning. Some people also mentioned that the exercises became a little repetitive after a while, especially in small groups, and that they felt unnatural. On average, the experience was rated as positive (Md 4 on the 5-point scale (1 – 5)).

The second question regarding the effects of the intervention was also answered with positive responses. Positive effects on the group were mentioned by most people interviewed. The sense of community reportedly increased and the group members increased their positive feedback on each other. Some quotations are as follows: “We have learned to listen more actively to each other”; “a common ground from which to see things has been developed”; “there is more energy in the group”; and “I have a new role as an educator, and we have lifted our work to a new level”. Emotional effects of the intervention included increased pleasure at work, a more positive view of each other, and an improved atmosphere in the group. Cognitive aspects reported by participants included descriptions of how everyday life includes many positive aspects, problems can be solved, and that it is better to focus on possibilities rather than on difficulties. One of the interviewees mentioned that the long-term absence due to illness had decreased after the intervention and another reported that the short-term absence had decreased. One frequent comment was that effects were observed after a short period of time and it would be interesting to see if they were maintained. At the same time, several interviewees told us that they continued to use the methods they had learned after the intervention period. The median of the interviewees’ ratings of the effects of the intervention was +3 on the scale from – 5 to +5.

The third question was about what we could have done to facilitate the intervention procedure for the managers. Twenty of 24 respondents answered “no” to this question. They reported that the instructions were clear and that the exercises were easy. One problem reported was insufficient time to complete the interventions due to heavy work load, and some interviewees expressed the wish to have had the chance to discuss issues related to the intervention with others who had completed it. One person argued that it would probably have been better if the project leaders had performed the interventions, and some thought that the exercises were boring during the 4^th^ and 5^th^ sessions. Another person thought the instructions to the exercises could have been more descriptive. Another suggestion was that a follow-up meeting be arranged for those who had completed the intervention.

## 5 Discussion

The aim with the present study was twofold; to present the development of an intervention model related to gratitude at workplaces and to investigate what effects this model might have. The present study showed that a five-session gratitude dialogue in groups of employees, supervised by their managers is feasible, is positively experienced by most of the participants and of the managers, and can enhance important parameters within organisations. Significant improvements were seen at assessment two (A2) in psychological well-being, engagement and job satisfaction for the intervention group, although with small effects. When looking at subgroups of work units, significant differences were shown for the units with “care of elderly and disabled” and “service” regarding engagement, job satisfaction and psychological capital.

Consistent with previous research, the gratitude intervention resulted in improved psychological health. This is a vital issue in the contemporary life of organisations because of mounting pressure on the workforce to produce, learn, adapt, and change in a fast-paced workplace. Indeed, stress and burn-out is one of our biggest work-related problems people face today [57]. Well-being at work and job satisfaction are two closely related concepts that affect overall performance at work [58].

To our knowledge, this is the first study to investigate the effect of a gratitude intervention on engagement and psychological capital and our results show that a gratitude intervention may enhance these aspects. Job engagement is associated with high levels of energy and strong identification with one’s work, and engagement predict job performance and client satisfaction [59]. Psychological capital is strongly related to numerous desirable employee characteristics in attitudes, behaviours, and performance at work. It is also negatively correlated to undesirable attitudes and behaviours [60]. According to DeMott et al. [61] PsyCap can also counteract stress in employees.

Using a control group can help to rule out any placebo effect of an intervention, whereby participants take part in similar, but not identical, activities as the intervention group. Wood et al. [5] have highlighted the need for this and sets a question mark after the strong results that gratitude interventions have depicted. In the present study, the control group was chosen to approximately match the intervention groups regarding work assignments, education and in terms of employment and assessments were made at the same time periods. However, the control participants were passive in that they did not participate in any comparable activity and this could be considered a weakness of this study.

The concept of training the trainer [62] is important in this context. The importance of the training is emphasized by the coaches/managers and that the trainers are accessible during the process. They also suggest follow-up sessions to support the coaches/managers in their efforts to keep the positive work environment. Wishes for a closer connection and more advice were expressed by the coaches. However, they also claimed that the model was easy to implement.

Another outcome of this study is that participants experienced the intervention as a positive experience and that, with the proper introduction and training, it could be implemented at the workplace without external involvement. Thus, our results indicate that this method is simple, enjoyable, and easy to implement. Among Japanese workers Komase et al. [63] performed a gratitude intervention program containing psychoeducation, gratitude lists and behavioral gratitude expression. They conclude gratitude interventions in organizations to be easy to understand and implement, time and cost efficient, have low dropout rates and they do not require experts in psychology.

Another feature of the intervention is that it was completed in groups rather than individually. In communicating mutual gratefulness at work, new dialogues and insights were revealed and created. This may have a transformative effect on the organisational culture such that a more positive view of work and co-workers emerges. Indeed, the qualitative results indicate that this was the case. The emotional closeness elicited by the gratitude dialogues can also increase a sense of psychological safety, which is an important trait of well-functioning groups [64].

The results of the present study showed that the intervention had better effects in some groups (like caring homes for elderly and persons with disability and service groups) than in others (like administration) (Table 2). The reason for this is difficult to speculate about. Maybe the group atmosphere was better from the beginning in some groups, maybe the coaches had a stronger position in some groups? And maybe these groups are relatively undernourished of the kind of attention they experienced here.

This study not only tested a certain method, but also addressed the subject of gratitude. Gratitude is about recognising the good elements of life and increasing this awareness. There is much evidence to suggest that we tend to see life in a darker light than necessary [65]. By focusing more on the aspects of life that we value, we can counteract our negativity bias and achieve a more balanced and positive view of reality. From the work of Lyubomirsky et. al. [66], we know that having a positive outlook on life is a fundamental aspect of good health, rewarding relationships, and thriving conditions at work.

One serious shortcoming of the present study is the dropout numbers. However, this is difficult to avoid in longitudinal, “real-life” studies. People quit their jobs, become sick, are busy with other things, and can become tired of the assessments. It is not clear whether the dropouts were incidental or systematic. However, following interviews with the managers, we believe that, for the main part, the dropout rate was incidental rather than systematic in the intervention group. The big differences of group sizes may also limit what conclusions that can be drawn. Unfortunately there is no information about age, gender and level of professional experience. Knowledge about those factors might have further increased our understanding.

To be noticed is that all the participants, mainly women, came from public organizations. No participants came from the private labour market, which may have affected the results.

We found positive effects of the interventions, and future work should aim to enhance the outcomes observed. From interviews with the managers, we received two suggestions on how to enhance and prolong the outcomes observed. One idea is that the interventions could be conducted by external consultants instead of the managers. An external specialist might be more convincing and more capable of leading the group sessions. This idea is supported by the results of our previous study, where two experienced psychologists conducted the groups [4]. In that study the effects were more pronounced than in the present study. Another idea is to have a follow-up session to support the managers in their continued quest for a positive work environment. In the present study, we found no lasting effect of the intervention at the 6-month follow up. This could suggest that it is necessary to engage in continual maintenance activity to secure a healthy working atmosphere. Without this, negativity bias could dominate the individual, the group, and the work environment. Another possible explanation for the lack of any long-term effect is the large number of dropouts prior to the third assessment. Like in all intervention studies in “real life” it is important to realize that there are a lot of factors that influence the course of life outside the context of the intervention.

There are several suggestions for future work. One concerns the intervention method. Combining the intervention method used in the present study with the self-guided gratitude journaling method used by Chan [38, 39] and Cheng et al. [40] could strengthen the effects of the intervention. This self-guided journaling technique is in accordance with Pennebaker and Seagal’s findings of significant effects of journaling on health and well-being [67]. In short, Pennebaker and Seagal’s theory proposes that humans have a need to form their history, that articulating this history in a narrative can help deal with trauma or injuring experiences, and that this can be generally considered as a coping mechanism. To write is a way to articulate, handle, and understand one’s own feelings and reactions in constructive ways. It is the writing itself, rather than discussion about the writing, that exerts this effect. Our need for comprehensive and realistic narratives could also be harnessed to develop and understand tasks and challenges at work. Gratefulness might be “another narrative”.

Another way to develop this intervention method could be by adopting a participant-directed method, by asking participants how they want to apply the basic ideas about thankfulness and positive psychology related to their experiences to the method. This could increase the applicability of the method to the actual organisation.

To expand the present research further another way would be to examine the relationship between the organisation and its customers/users. For example, future studies could investigate whether customers/users get better products or care if the staff or personnel feel better or have a greater psychological capital. There are reasons to believe that this is the case. In the present study, we did not measure the effect of intervention on customer satisfaction. There is evidence that gratitude can increase prosocial behaviour [24], organisational citizenship behaviour [25, 26], and a sense of responsibility [27]. It seems logical that these factors can have a positive spill-over effect on customer satisfaction. To our knowledge, no study has yet demonstrated there to be a direct empirical link between gratitude and customer satisfaction. Our results indicate that gratitude interventions can improve employee well-being, job satisfaction, engagement and, to a lesser degree, psychological capital. Previous research has found links between employee well-being and service quality [68] and customer satisfaction [69], job satisfaction and customer satisfaction [70–72], engagement and customer loyalty [73] and customer satisfaction [74], positive psychological capital and customer orientation [75], and service quality and customer satisfaction [76]. Consequently, we have good reason to believe that gratitude interventions have a positive impact on customer satisfaction, but a direct link remains to be shown.

### 6. Conclusion

In conclusion, our results suggest that gratitude dialogues at work may be an effective way of improving employee wellbeing. The study showed that a five-session gratitude dialogue in groups of employees, supervised by their managers is feasible, is positively experienced by most of the participants and the managers, and can enhance important parameters within organisations. The results showed that the intervention improved the psychological well-being, engagement and job satisfaction.

## Data Availability

The data that support the findings of this study are available on request from the corresponding author, [AB]. The data are not publicly available due to restrictions [e.g. their containing information that could compromise the privacy of research participants].
